# Patterns of Use of e-Cigarettes and Their Respiratory Effects: Protocol for an Umbrella Review

**DOI:** 10.2196/60325

**Published:** 2024-09-04

**Authors:** Giusy Rita Maria La Rosa, Riccardo Polosa, Renée O’Leary

**Affiliations:** 1 Department of Clinical and Experimental Medicine University of Catania Catania Italy; 2 Center for the Acceleration of Harm Reduction University of Catania Catania Italy

**Keywords:** dual use, electronic nicotine delivery systems, naïve use, respiratory, smoking cessation, umbrella review

## Abstract

**Background:**

Electronic nicotine delivery systems (ENDS)—e-cigarettes or vapes—have been shown to substantially reduce or eliminate many toxins compared with cigarette smoke, but simultaneously ENDS use also produces their own unique toxins. Yet the patterns of use among people who use ENDS are not homogeneous. Some people who use ENDS also smoke cigarettes (dual use). Other people who formerly smoked cigarettes are completely substituting ENDS (exclusive use). A small number of people who have never smoked cigarettes are using ENDS (naïve use of nicotine). Each of these patterns of use results in different exposures to toxins. Unfortunately, epidemiological studies routinely group together any ENDS use regardless of other tobacco use.

**Objective:**

This umbrella review primarily aims to present all the evidence available on the respiratory effects of ENDS use by adults based on their pattern of use: dual use, exclusive use, and naïve use. With each of these patterns of use, are there benefits, no changes, or harmful effects on respiratory functioning? Our objective is to provide clinicians with a detailed analysis of how different patterns of ENDS use impact respiratory functioning and to point to the best sources of evidence.

**Methods:**

This umbrella review follows the Methods for Overviews of Reviews framework and the PRIOR (Preferred Reporting Items for Overviews of Reviews) statement. Systematic reviews published since 2019 will be searched across 4 databases and 3 gray literature sources. Additional searches will include citation chasing, references lists, and referrals from respiratory specialists. The quality of included reviews will be evaluated using the AMSTAR2 (A Measurement Tool to Assess Systematic Reviews) checklist. We will document biases in 3 areas: protocol deviations, biases from the Oxford Catalogue of Bias, and internal data discrepancies. Two reviewers will independently conduct the search and quality assessments. Our analysis will focus on reviews rated as moderate or high confidence by AMSTAR2. We will use the Vote Counting Direction of Effect method to manage expected data heterogeneity, assessing whether ENDS use is beneficial or detrimental, or has no effect on respiratory functions based on the pattern of use.

**Results:**

The review is expected to be completed by December 2024. The database search was concluded in April 2024, and data extraction and bias assessment were completed in June 2024. The analysis phase is planned to be completed by October 2024.

**Conclusions:**

A thorough and comprehensive assessment of the evidence will better inform the contentious debate over the respiratory effects of ENDS providing much needed clarity by linking their effects to specific usage patterns. This analysis is particularly crucial in understanding the risks associated with continued cigarette smoking.

**Trial Registration:**

PROSPERO CRD42024540034; https://www.crd.york.ac.uk/prospero/display_record.php?RecordID=540034

**International Registered Report Identifier (IRRID):**

DERR1-10.2196/60325

## Introduction

### Background

Electronic nicotine delivery systems (ENDS)—e-cigarettes or vapes—have been shown to substantially reduce or eliminate many toxins compared with cigarette smoke [1,2]. Although limited in number, ENDS use does produce their own unique toxins, such as metal exposure [3], and others that are present in tobacco smoke, such as carbonyls, although at much lower levels [4]. In terms of risk, most e-cigarette analyses show the cancer potencies of ENDS to be less than 1% of that of cigarette smoke [5]. Furthermore, the lung cancer risk from vaping is estimated to be 5 orders of magnitude lower than smoking [6].

Yet the patterns of use among people who use ENDS are not homogeneous. Some people who use ENDS also smoke cigarettes (dual use). Other people who formerly smoked cigarettes are completely substituting ENDS (exclusive use). A small number of people who have never smoked cigarettes are using ENDS (naïve use of nicotine). Unfortunately, epidemiological studies routinely group together any ENDS use regardless of other tobacco use [7], and this grouping together of populations with different patterns of use confounds the findings of studies [3].

For each of these discrete groups, ENDS exposure will have differing effects on their respiratory health and functioning. In dual use, ongoing exposure to tobacco smoke, even from as few as 1 or 2 cigarettes a day, continues to generate high risks for tobacco-related diseases [8-10]. Daily use of cigarettes and less than daily use of ENDS is the most common pattern of dual use, but there are differing patterns [11,12]. For exclusive use, smoking history (smoking career) may have already impacted respiratory functioning [9], regardless of the subsequent reduction in exposure to toxins. Advocates of tobacco harm reduction stress complete abstinence from cigarettes to obtain potential benefits from ENDS use [3]. Although the numbers of nicotine-naïve individuals using ENDS are small (for example, 0.6% to 0.7% of English adults who never smoked use ENDS [3]), evidence on the respiratory effects of ENDS use by these individuals is crucial for their health care and their lifestyle choices. Data on the effects of naïve use contribute to the research by identifying potential risks or harms of vaping that are not a consequence of prior smoking history [3].

Numerous systematic reviews have examined the respiratory effects of ENDS use with varying conclusions [13-16]. For instance, one systematic review found that vaping increases sensitive measures of airway resistance but does not appear to affect standard measurements of lung function test (eg, forced expiratory volume in 1 second [FEV1], forced vital capacity [FVC], or the FEV1/FVC ratio) [14]. However, this review primarily focused on the acute effects of vaping. Another review by Alqahtani et al [15] highlighted the heterogeneity and inconsistencies in existing studies, underscoring the need for further research using robust study designs. Given the diverse findings and methodological differences in the existing literature, conducting an umbrella review is essential for a comprehensive and critical synthesis of the current evidence.

### Research Question

The primary purpose of our umbrella review is to present all the evidence available on the respiratory effects of ENDS use by adults based on their pattern of use: dual use with continued cigarette smoking, exclusive use after abstaining from cigarettes, and use by adults who have never smoked cigarettes. With each of these patterns of use, are there benefits, no changes, or harmful effects on respiratory functioning? Our secondary purpose is to identify the higher quality systematic reviews through a rigorous assessment of both their conduct and reporting. Our objective is to provide clinicians with a detailed analysis of how different patterns of ENDS use impact respiratory functioning and to point to the best sources of evidence.

## Methods

### Overview

Our umbrella review was developed with the Methods for Overviews of Reviews (MOoR) framework [17,18] and the PRIOR (Preferred Reporting Items for Overviews of Reviews) statement [19].

### Population, Intervention, Comparator, and Outcomes Criteria

The PICO (Population, Intervention, Comparator, and Outcome) criteria below define the scope of our umbrella review.

- Population: adults (≥18 years old) who smoke cigarettes, adults who have quit smoking, and adults who have never smoked (<100 cigarettes lifetime)
- Intervention: ENDS use
- Comparator: within-subject changes, control group or arm (including placebo), or longitudinal cohort
- Outcome: any self-reported or clinically measured change in respiratory function

Respiratory function outcomes include both respiratory symptoms and test measurements. The symptoms include breathlessness, dyspnea, breathing difficulties, wheeze, cough, sputum, and phlegm. Tests include spirometry (ie, FEV1, FVC, forced expiratory flow at 25%-75% of forced vital capacity, peak expiratory flow, and FEV1/FVC%), airway resistance, impulse oscillometry, impaired mucociliary clearance, and lung function (ie, total lung capacity, residual volume, and expiratory reserve volume). Other outcomes include but are not limited to computed tomography findings of emphysema, airway remodeling, and small airway loss; respiratory-related quality of life and exercise limitations; incidence or prevalence of respiratory disease; and exacerbations of previous respiratory disease. Finally, outcomes include health care resource utilization for respiratory disease–related ambulatory care, emergency department visits, and hospitalization [14]. Other respiratory outcomes not in this list will be added as reported. E-cigarette or vaping use–associated lung injury (EVALI) data, if available, will be evaluated as a separate class of outcome.

### Search and Selection Processes

Databases for the search include Scopus (Elsevier), MEDLINE (via PubMed), Cochrane Database of Systematic Reviews, International Prospective Register of Systematic Reviews (PROSPERO), and Epistemonikos, and the gray literature databases MedNar, National Technical Information Service, and WorldWideScience.org.

We will include systematic reviews published from 2019. Restricting the time frame is justified by 2 main reasons. First, these systematic reviews included the latest primary studies conducted with the most advanced devices. Second, these devices have evolved significantly over time, leading to considerable changes in their designs, making older models outdated and no longer available in the market [20,21]. A newer style model is the single-use disposables that were introduced to the market in 2019 [22]. The modifications in newer models have played a role in reducing exposure to silicon and solder [21], while alterations in ENDS liquids exhibit potential for lowering carbonyl emissions [23]. Therefore, it is imperative that our findings and conclusions are grounded in the most up-to-date evidence derived from tests conducted with these newer models.

Terms such as “electronic nicotine,” “e-cigarette,” and “vaping” will be combined with respiratory-related keywords such as “respiratory,” “lungs,” and “pulmonary” using Boolean operators (ie, OR for synonyms and AND for combining intervention and outcome). Title, abstract, and keywords fields will be selected for the search. For each database, the search terms and syntax will be adjusted according to the database-specific requirements, such as MeSH (Medical Subject Headings) terms for PubMed. A filter for detecting only systematic reviews will be applied where possible. As an example, the search strategy for the Scopus database is as follows: TITLE-ABS-KEY ( (“e-cig*” OR “vaping” OR “e-cigarette” OR “vapers” OR “Electronic nicotine delivery systems”) AND (“respiratory” OR “pulmonary” OR “lungs” OR “breathlessness” OR “dyspnea” OR “wheeze” OR “cough” OR “sputum” OR “phlegm” OR “spirometry”) AND (“systematic review” OR “meta-analysis”) ) AND PUBYEAR > 2018 AND PUBYEAR < 2025. Each search will be meticulously documented by screenshots of the database pages displaying the search syntax and the number of results, and all retrieved records will be exported into an EndNote library, where duplicates will be identified and removed. Two researchers will independently screen the records for all PICO criteria in the title and abstract (or summary); publications lacking any PICO criterion will be excluded. Discrepancies in the exclusions will be decided by the project leader, and the interrater agreement reported.

After the database searches are completed, we will conduct a second round of secondary searches. One search will be a citation chase (snowball search) in Google Scholar. The second search will be a check of the references of the included systematic reviews. These 2 searches will be conducted by 2 reviewers independently. Secondary search publications will be included or excluded based on the full paper criteria. Finally, the list of included systematic reviews will be checked by two experts in respiratory diseases.

### Inclusion and Exclusion Criteria

After the title and abstract exclusion process, a full paper review will be conducted by 2 researchers independently for the inclusion and exclusion criteria shown in Table 1.

**Table 1:** Inclusion and exclusion criteria.^a^

Criteria	Inclusion	Exclusion
Article type	Systematic review with a minimum search of 2 databases	All other study designs
Language	All languages	Reviews for which translation is unavailable
Publisher	Academic journals Government reports Medical organizations	Predatory journals (not indexed in PubMed or Directory of Open Access Journals) Conference abstracts
Primary study designs	Clinical trials (randomized or nonrandomized) Experimental studies Longitudinal cohort studies	Cross-sectional study data Surveys Case studies Qualitative studies Animal studies In vitro studies
Population	Adults who smoke cigarettes, adults who have quit smoking, and adults who have never smoked	Youth
Data	Changes in disease symptoms Clinical test measurements Self-reported health status	Passive or second-hand exposure Only 1 primary study from included study designs
Bias assessment of primary studies	Individual primary studies assessed with any method	No bias assessment of individual primary studies Inappropriate bias tool
Analysis-synthesis method	Meta-analysis Tabulation Narrative assessment	Solely summary of individual primary studies

^a^Studies excluded at the full paper examination will be listed in an appendix and will note the reason for exclusion.

### Data Extraction

For efficiency, the data extraction and quality assessments of a systematic review (including its supplementary materials and protocol) will be conducted concurrently.

The specific items for data extraction will be chosen by the research team and pilot-tested on 2 systematic reviews. The planned data items are included in Textbox 1. For systematic reviews that include multiple study designs, data will be selected solely from those that fulfill the inclusion criteria. One researcher will perform the data extraction, and a second reviewer will 100% cross-check it.

**Data items for the data extraction.**

- Bibliographic information
- Funders and conflicts of interest
- Databases searched and dates
- Secondary searches performed
- Inclusion-exclusion criteria of the systematic reviews
- Population demographics
- Intervention description including the electronic nicotine delivery system device and nicotine strength
- Outcome definition and measurement
- Primary studies with their bias assessments
- Narrative assessment (if applicable)
- Data analyses (including sensitivity analyses)
- Meta-analyses
- Any subgroup analyses
- GRADE (Grading of Recommendations, Assessment, Development, and Evaluations) or authors’ assessment of confidence in the evidence
- Limitations identified by the systematic review authors
- Conclusions quoted

### Quality Assessment

We will use the AMSTAR2 (A Measurement Tool to Assess Systematic Reviews) checklist [24] to assess the quality of the systematic reviews and identify the higher quality ones. The review team will designate AMSTAR2 item 7 (a list of excluded studies) as a *noncritical weakness* and not a *critical flaw* because PRISMA (Preferred Reporting Items for Systematic Reviews and Meta-Analyses) [25], a widely used reporting standard, does not require an excluded studies list. Two reviewers will independently complete the checklist, and the project leader will decide any unresolved discrepancies. For the overall confidence rating, the project leader will score its confidence rating as *high*, *moderate*, *low*, or *critically low* as per the AMSTAR2 criteria except for item 7 as noted above.

A second quality assessment of the reviews will be an examination for reporting biases in 3 areas. The first will be unreported deviations from the protocol. The second will be a checklist of reporting biases drawn from the Oxford Catalogue of Bias [26]: spin bias of nonsignificant findings, omitted findings, one-sided reference bias, framing by over- or underemphasis of outcomes, and overreliance on *P* values (as compared with clinical relevance). The third examination will check for internal data reporting discrepancies within the systematic review publication with the checklist proposed by Puljak et al [27].

We expect that this umbrella review will include a systematic review of ENDS substitution of cigarette smoking that we published with coauthors [16]. To preclude the possibility of bias, 2 neutral third-party reviewers will conduct the AMSTAR2 and reporting bias assessments for this systematic review.

The search processes and results will be displayed in a PRIOR flow diagram. A study table will report the systematic reviews, a second table will display their AMSTAR2 scoring and rating, and a third table will record reporting biases. A citation matrix will list all the primary studies included in the systematic reviews. Analyses will be presented based on the pattern of use: dual use, exclusive use, and naïve use. Subgroup analyses are planned for populations with asthma or chronic obstructive pulmonary disease.

### Planned Analyses

In our review, heterogeneity presents a challenge to select the appropriate method of analysis. The evidence base of primary studies is expected to be highly heterogeneous. Particularly notable are the variations in ENDS devices, nicotine strengths, the duration of the intervention, and the duration of follow-up. Heterogeneity occurs also between the systematic reviews, primarily from differences in their inclusion and exclusion criteria. Heterogeneity also arises from earlier and later search dates resulting in different primary studies analyzed in the systematic reviews.

For these reasons, a statistical meta-analysis will most likely not be feasible. Our analysis method will be a Vote Counting Direction of Effect, which is appropriate for heterogeneous data [28]. This analysis will indicate if ENDS use is beneficial, detrimental, or has no effect on respiratory functions. To reduce the risk of bias for the synthesis, only systematic reviews rated at AMSTAR2 moderate or high confidence will be analyzed. The GRADE (Grading of Recommendations, Assessment, Development, and Evaluations) or bias description rating of the findings made in the systematic reviews will be included in the Vote Counting Direction of Effect.

As a component of our analyses, we will calculate the overlap of primary studies between the systematic reviews. This analysis is necessary to determine if certain primary studies are overrepresented and therefore would result in these studies being overweighted in the findings. We will apply the *corrected coverage area* calculation [29] to determine which, if any, primary studies could skew the findings of our umbrella review.

Respiratory functioning outcomes (see PICO above) will be ranked in importance for clinical care by a respiratory physician.

A sensitivity analysis will compare findings in the analyses with those from the systematic reviews rated at low confidence. If necessary, a sensitivity analysis will compare results from commercially funded systematic reviews (including tobacco industry, vape companies, pharmaceutical companies, and medical equipment manufacturers) with all others.

The observations of reporting biases will be reported in a table. The impact of reporting biases on the research literature will be explored in the Discussion and will contribute to our recommendations for future research

If 10 or more systematic reviews are included, publication bias will be investigated with a tabulation of the conclusions of the systematic reviews on the respiratory effects of ENDS usage.

Any deviations from the protocol will be reported in the published umbrella review.

## Results

The protocol has been registered in PROSPERO (CRD42024540034), and the umbrella review is expected to be completed by December 2024, followed by the submission of the review for journal publication. The database search was concluded in April 2024, and data extraction and bias assessments were completed in June 2024. The analysis phase is planned to be completed by October 2024.

## Discussion

This will be the first umbrella review to provide a comprehensive overview of respiratory symptoms in ENDS users. Based on the current evidence, exclusive ENDS use is expected to significantly improve respiratory outcomes after completely switching away from cigarette smoking. In contrast, dual use with concurrent tobacco cigarette consumption may experience less improvement than exclusive ENDS use. Additionally, we anticipate a deterioration or no effect on respiratory symptoms in naïve use of vapes.

This umbrella review is expected to have a potential significant impact thanks to its methodological rigor, grounded in the MOoR framework and the PRIOR statement. However, the scarcity of data on the respiratory health effects of ENDS in never smokers is a possible limitation that may affect the robustness of the study findings.

Methodological rigor is crucial in a highly polarized research field [30-32]. A rigorous and comprehensive evaluation of the evidence will clarify some of the key controversies surrounding the respiratory impacts of ENDS, correlating their effects with specific usage patterns, particularly the risks associated with ongoing cigarette smoking.

The intention is to widely disseminate the publication of the study protocol and the completed review through articles in peer-reviewed journals and conference presentations. The link to the publication will be shared via our email list to hundreds of academic experts in the field and to general media with a press release. Dissemination will also occur via social media platforms.

A summary of the results will be available on the study website for public access. The anonymized data will be available to researchers on reasonable request.

## Abbreviations

AMSTAR2: A Measurement Tool to Assess Systematic Reviews
ENDS: electronic nicotine delivery systems
EVALI: e-cigarette or vaping use–associated lung injury
FEV1: forced expiratory volume in 1 second
FVC: forced vital capacity
GRADE: Grading of Recommendations, Assessment, Development, and Evaluations
MOoR: Methods for Overviews of Reviews
PICO: Population, Intervention, Comparator, and Outcome
PRIOR: Preferred Reporting Items for Overviews of Reviews
PRISMA: Preferred Reporting Items for Systematic Reviews and Meta-Analyses
PROSPERO: International Prospective Register of Systematic Reviews

## Data Availability

Data sharing is not applicable to this study as no datasets were generated or analyzed during this study.
